# Sternal Reconstruction After Cardiac Transplantation: A Case of an Oversized Donor Heart

**Published:** 2012-01-24

**Authors:** Hamid R. Zahiri, Amy Stump, Shahrooz Kelishadi, Alexandra Condé-Green, Ronald P. Silverman, Luther Holton, Devinder P. Singh

**Affiliations:** ^a^Division of General Surgery, Department of Surgery, University of Maryland School of Medicine, Baltimore; ^b^Division of Plastic and Reconstructive Surgery, Department of Surgery, University of Louisville School of Medicine, Kentucky; ^c^Division of Plastic and Reconstructive Surgery, Department of Surgery, University of Maryland School of Medicine, Baltimore

## Abstract

**Background:** We present a unique case of a cardiac transplant recipient who received an oversized heart. **Methods:** To allow the chest to accommodate the organ, extensive resection of the bony chest wall was performed. As both pectoralis major myocutaneous flaps and omental transposition were insufficient to cover the wound, a chest rotational flap was chosen. **Results:** The large size of the flap allowed us to cover the entire protuberant heart, and the excess soft tissue absorbed the pulsations from the heart without placing tension on the suture line. **Conclusion:** While the closure of complex sternal wounds can pose great challenges, the plastic surgeon possesses a variety of options including pectoralis, omental, rectus abdominus, latissimus dorsi as well as skin and subcutaneous flap closures to choose from.

More than 2200 heart transplants were performed in the United States in 2007.^1^ With mediastinitis affecting about 7.5% of cardiac transplant patients, the plastic surgeon practicing in an institution where heart transplants are performed can expect occasional consultation for sternal reconstruction. Whereas the reconstructive surgeons are often called upon to fill large mediastinal tissue cavities, occasionally patients present with a heart that is too large for the chest. These cases are challenging and often require a different reconstructive approach. The unique characteristics of cardiac transplant patients require the plastic surgeon to have a large repertoire of treatments to fit the clinical scenario. We present here the case of a cardiac transplant patient whose unique wound characteristics required us to move from common to more unusual treatment options in order to effectively perform sternal reconstruction.

## CASE

**Figure 1:**
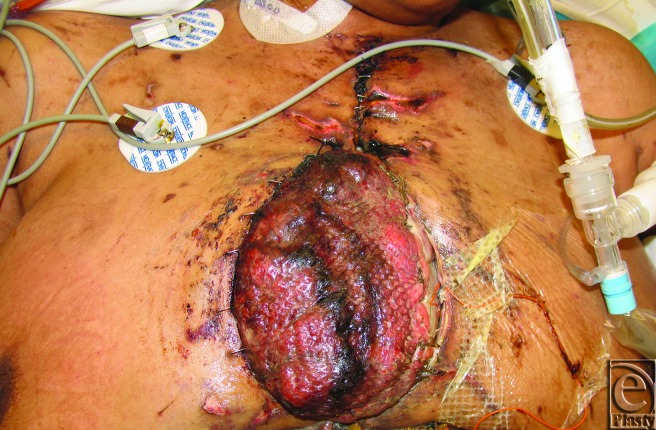
Projection of the heart above bony chest wall posttransplantation.

A previously healthy 42-year-old woman developed acute heart failure consistent with postviral cardiomyopathy. She developed cardiogenic shock and was emergently transferred to the University of Maryland's Cardiac Surgery Division for biventricular assist device placement. Her hospital course became complicated by VAD infection requiring long-term antibiotics and adrenal insufficiency necessitating chronic steroid therapy. Once stabilized, she was listed for heart transplantation, which she received 1 year after initial presentation. During her transplantation, the donor heart was found to be significantly larger than the patient's own heart, resulting in difficulty placing it within her relatively shallow pericardial sac. After implantation, the donor heart projected 8 cm above the bony chest wall and herniated nearly completely from the mediastinum with each ventilator inspiration (Fig 1, **Video Link**). The patient with the sternal retractor left in place and a silo of GoreTex placed over the implanted heart was transferred back to the cardiac ICU. One day after transplantation, the patient developed right-sided heart failure necessitating placement of a right ventricle assist device (RVAD). Once she stabilized, the Division of Plastic Surgery was consulted for chest wall reconstruction.

**Figure 2:**
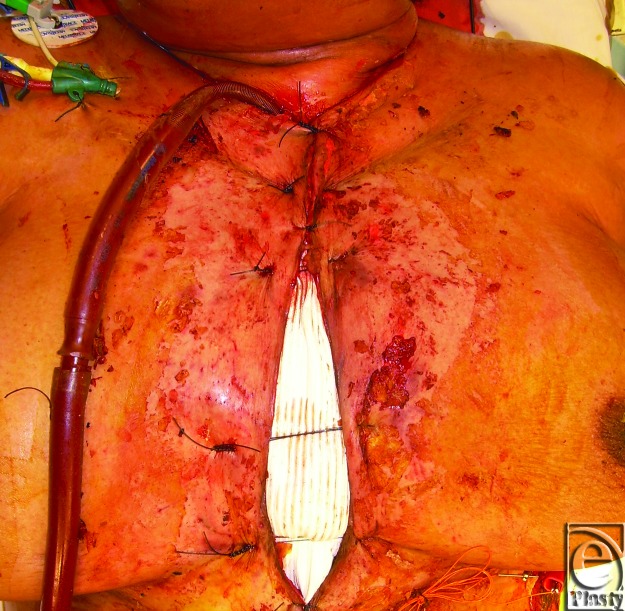
Myocutaneous advancement flap and DualMesh Plus closure of sternal defect.

Four days after transplantation, the patient was taken to the operating room. The objective was to protect the heart until reexploration of her mediastinum for RVAD removal. Thus, resection of the entire sternum along with partial resection of 5 ribs bilaterally was performed. Nevertheless, the heart still protruded above skin level. Bilateral pectoralis major myocutaneous advancement flaps were raised and transferred to the midline. Attempts to mechanically close pectoralis flaps resulted in elevated pulmonary artery and central venous pressures. Therefore, a sheet of DualMesh Plus (GoreTex) was sutured to the chest wall to cover the heart followed by superior and inferior closure of the pectoralis flaps. As possible, the skin was closed over the defect and an additional piece of Dual Mesh Plus was used to cover a 4-cm midline cutaneous defect (Fig 2).

**Figure 3:**
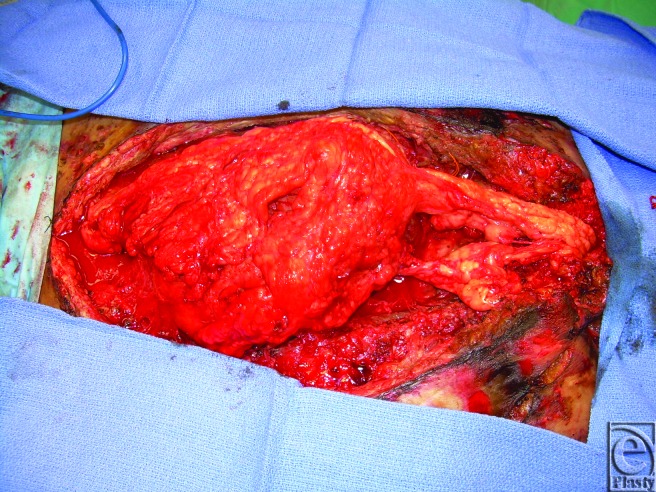
Omental flap coverage of exposed heart through midline defect.

When the patient's cardiac status improved, RVAD was removed and definitive closure was attempted 11 days after cardiac transplantation. The DualMesh Plus was removed and the skin opened. The omentum was harvested and rotated to cover the defect where the pectoralis muscle could not be closed at the midline (Fig 3). The skin was closed, and a split-thickness skin graft was placed on top of the extra-abdominal omental flap to cover the defect.

Five weeks after transplantation, she developed infection of her sternal wound. Debridement of the sternal wound created a 150 cm^2^ defect in her anterior chest wall. A skin and subcutaneous flap approximately 100 cm^2^ in size was advanced from the left chest. The small remaining defect was closed with a vacuum-assisted closure device. Over the next week, cardiac pulsations caused the omental flap to pull apart from the skin on the patient's left side, leaving exposed heart. Few options for reconstruction remained at this point. Therefore, a large rotational flap from the left chest, including the entire left breast as well as the pectoralis major muscle superiorly, was elevated and advanced to cover the protruding heart. This left a substantial wound defect in the inferior and right aspects of the heart. Thus, the right pectoralis myocutaneous flap was completely separated from its remaining costal attachments laterally to the anterior axillary line and from the clavicle superiorly and advanced medially as a myocutaneous flap to cover the remaining defect. The left chest donor site was closed by advancing skin from the lateral chest wall.

**Figure 4:**
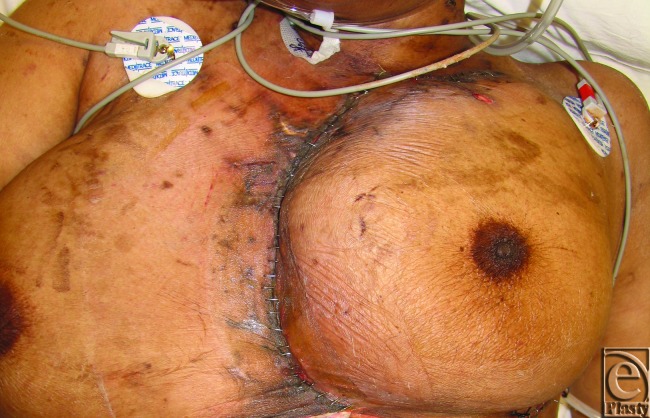
Closure of sternal defect using a large rotational flap from the left chest.

This final procedure proved sufficiently durable coverage for the heart (Fig 4). The patient had no further issues with her sternal wound and was discharged to a rehabilitation facility 3 months after her transplantation.

## DISCUSSION

Infection typically causes sternal wound breakdown in the setting of cardiac transplantation, often potentiated by immunosuppressant use. In this patient, wound healing was impaired by immunosuppressants and continual mechanical trauma to the wound caused by the beating oversized heart.

Our initial treatment for this patient included a pectoralis major myocutaneous flap. The pectoralis major muscle flap is often the first choice in sternal reconstruction due to its consistent vascular anatomy and close proximity to the wound. In addition, a pectoralis flap can be transferred onto the sternal wound either as a turnover or as an advancement flap.^2^ As a turnover flap, the muscle's attachment to the humerus is divided and the muscle is turned over lateral to medial into the wound. As an advancement flap, the muscle is dissected off of the chest wall and is transferred into the sternal wound, with or without overlying skin.

When pectoralis flaps proved insufficient, omental transposition and overlying split-thickness skin graft were used to protect the heart. The omental flap is effective for filling a large of amount of dead space.^3^^-^7 The omentum is generally harvested from the transverse colon through a midline incision in the upper abdomen.^3^ A well-vascularized region of the omentum is advanced onto the wound utilizing a passage made through the diaphragm in front of the pericardium.

When the omental flap became infected, we used a chest skin rotational flap based on the size, viability, and proximity of the tissue to the wound. The large size of the flap allowed coverage of the entire protuberant heart, and the excess soft tissue absorbed the pulsations from the heart without placing tension on the suture line. This flap proved to be an effective surgical treatment.

Other options for adjacent tissue transfer in sternal reconstruction include rectus abdominus, latissimus dorsi, and free tissue transfer.^8^^-^12

## CONCLUSION

We present here the unique case of a cardiac transplant recipient who received an oversized heart. To allow her chest to accommodate the organ, she underwent extensive resection of the bony chest wall. As both pectoralis major myocutaneous flaps and omental transposition were insufficient to cover the wound, the decision was made to use a chest rotational flap, which was efficacious.

While the closure of complex sternal wounds can pose great challenges, the plastic surgeon possesses a variety of options including pectoralis, omental, rectus abdominus, latissimus dorsi as well as skin and subcutaneous flap closures to choose from.
